# How Personal Perspectives Shape Health Professionals’ Perceptions of Fetal Alcohol Spectrum Disorder and Risk

**DOI:** 10.3390/ijerph16111936

**Published:** 2019-05-31

**Authors:** Kerryn Bagley, Dorothy Badry

**Affiliations:** 1 Rural Health School, La Trobe University, Melbourne, VIC 3086, Australia; 2 Faculty of Social Work, University of Calgary, Calgary, AL T2N 1N4, Canada; badry@ucalgary.ca

**Keywords:** fetal alcohol spectrum disorder, FASD, non-clinical factors, alcohol, pregnancy, risk

## Abstract

This article examines how health, allied health and social service professionals’ personal perspectives about alcohol and the risks associated with alcohol consumption become non-clinical factors that may influence their professional practice responses in relation to fetal alcohol spectrum disorder (FASD). It presents findings derived from a qualitative, interview-based study of professionals from a range of health, allied health and social service professions in New Zealand. The data derived from these interviews revealed four frames of reference that practitioners use when thinking about alcohol and risk: reflection on personal experience; experiences of friends, relatives and colleagues; social constructions of alcohol use and misuse; and comparisons to other types of drug use. The article concludes that these non-clinical factors are important considerations in professional decision making about FASD.

## 1. Introduction

Fetal alcohol spectrum disorder (FASD) is a life-long condition caused by prenatal exposure to alcohol. It is characterized by a range of neurological and physical impairments that can affect cognition, day to day functioning, learning and behavior [1], and can have deleterious effects on an individual’s overall health and wellbeing. However, it is a condition that has been poorly understood historically, and which has only recently emerged as a priority concern in global health outcomes. As a result, internationally, there are inconsistencies in how FASD is identified, managed, prevented and understood, leading to quite widespread practice differences [2,3].

Without appropriate support, people with FASD are at high risk of adverse psychosocial outcomes including poor mental health, involvement with the justice system and early disengagement from the education system [4]. While many professionals are broadly aware that alcohol use and pregnancy can constitute health risks, most have received no formal or professional training on this topic [5,6,7]. Meanwhile, public discourses around alcohol in society are at times contradictory or counter-productive—for example, Hill et al. identify the challenges faced by young adults in a world where “everything is telling you to drink” [8]. Where FASD is concerned, health, allied health and social service professionals often work in a climate of incomplete information [9,10], and this can result in the formation of professional views that are informed in part by personal experience or opinion. Furthermore, where prenatal alcohol consumption is concerned, professionals’ experiences and opinions are connected to, and may have implications for, how risks are understood. These two issues can potentially affect the characteristics of health and social service support for FASD, and are therefore deserving of investigation.

The term ‘non-clinical factors’ is identified in this research as personal viewpoints, experiences, observations, beliefs and values in relation to alcohol use that are held by individuals. For comparison, Cameron identifies ‘common factors’ in human services as inclusive of the characteristics of the professional, client and various systems in which practice is carried out. Cameron further unpacks the complexity of ‘change work’, and recognizes that the practitioner is a human being whose experiences influence their professional responses and that “the determination of the choice of a particular path must be shaped substantially by the person of the practitioner” [11] (p. 158). Professional practice standards in response to FASD do not exist in most disciplines, and no mandatory training is required in human service settings. In Alberta, Canada, an FASD Community of Practice was developed in response to meeting the needs of children and families involved with the child welfare system [12]. Extensive work was undertaken to offer training to case workers and caregivers on FASD, including foster parents and guardians, because the intense nature of effectively caring for children with FASD is underestimated [13]. It was determined that the level of engagement of caseworkers including regular contact had a positive influence on placement stability [14]. FASD is a complex disability that intersects with the fields of health, ethics, justice, philosophy and science, and requires sound clinical knowledge. Social workers in particular are bound to encounter individuals with FASD in practice, given estimations that over 20% of children in care have FASD [15].

Extant research on health and allied health professional practice demonstrates that personal perceptions interact with professional decision making across various fields. As Hajjaj et al. note, while evidence-based medicine and diagnostic protocols provide a firm clinical basis for decisions, these also interact with ‘non-clinical’ influences. These non-clinical influences include both client and practitioner factors. For practitioners, examples of non-clinical factors include personal characteristics like faith or culture, time constraints, availability of resources among others [16]. Similarly, research in physiotherapy by Smith et al. has recognized various practitioner factors in clinical decision making. Their model for understanding practitioner factors includes four key elements: a multidimensional knowledge base, a practice model, a sense of identity and personal frames of reference [17].

It is important to note that these studies do not frame non-clinical influences as inherently negative: many of them emphasize the benefits that practitioners’ non-clinical influences can bring to effective health care, including increased empathy and, for example, openness to shared decision making [18]. However, a particular challenge where FASD is concerned is that there can be a significant gap in professional knowledge [19] and/or disagreement about the evidence base for appropriate responses to FASD, leading to inconsistent health or diagnostic outcomes, as well as inconsistent preventative advice about the presumed ‘safe’ levels of prenatal alcohol consumption [20]. For illustration, Smylie and Ussher [21], point out the polarization that exists between child protection and substance use services where child safety and women’s substance use/health are often treated dichotomously. Badry and Choate [19] argue that FASD is one of most complex challenges in social work today, particularly given multiple practice environments such as health, child welfare, education, family and criminal courts, mental health, homelessness, substance use treatment, trauma and prevention work.

Research focused specifically on professionals’ engagements with and responses to FASD indicate that professionals, in general, lack appropriate advice and support [22]. In one example, research demonstrated that physicians’ personal attitudes about alcohol can affect their willingness to even consider screening for or identifying FASD as a health issue [23]. Other studies have revealed that professionals across a broad range of helping professions are either unaware of FASD or not sufficiently familiar with it to be able to offer appropriate support [6,7,24,25,26]. Coons et al. [27] suggest that providing advice on alcohol use and pregnancy is neither routine or consistent in health care practice, and that embedding clear, balanced and consistent messaging within clinical practice is a critical aspect of prevention. However, as they elsewhere note [28], providing such advice is further challenged by varying levels of knowledge, education and training on FASD, as well as personal beliefs regarding women’s autonomy and pre-existing beliefs and subjective biases regarding who uses alcohol during pregnancy. It is recognized that the topic of alcohol use and pregnancy can be uncomfortable for health care providers to address, and confusion exists about whose role it is in the health care system to respond to this concern.

As Bagley has previously described [29], these circumstances can result in practitioners’ personal understandings of the effects and risks of prenatal alcohol consumption having a presence in their professional decision making. These interpretations are often informed by prevailing social attitudes towards alcohol consumption (or abstinence), as well as assumptions about what segments of the population may be at greater or lesser risk of harm associated with alcohol. As Webb notes, such constructions of risk may reflect other sorts of social or cultural biases borne out in public opinion [30]. With these complexities in mind, this paper examines medical, health and social service professionals’ attitudes towards alcohol, risk and FASD in order to understand the possible scope of these personal perceptions, and to consider how these views might then influence professional decision making.

## 2. Materials and Methods

### 2.1. Study Context

The findings presented in this article are based on data collected for a larger qualitative research project investigating New Zealand health, allied health and social service professionals’ knowledge and practices concerning FASD [31]. A previously published article from this project [29] broadly investigated practitioner knowledge of and responses to FASD. The present article focusses more specifically on professionals’ attitudes and assumptions about prenatal alcohol consumption. The authors’ engagement with this topic reflects their own professional standing as social workers and social work academics who have experience working with FASD from professional, personal and scholarly perspectives. This positioning has informed their approach and their interactions with the research participants—all health, allied health and social service professionals. The research focus arose organically from the larger project as a consequence of professionals reflecting on their personal attitudes towards alcohol and risk. In particular, participants reflected on the prevalence of alcohol in their social environment and compared this with their perception of alcohol consumption as informed by their professional knowledge. This led to many participants articulating a sense of disjuncture, which became the catalyst for further investigation.

### 2.2. Study Design

This study was grounded in an ethnographic research paradigm that sought to foreground the voices and experiences of the research participants. The data collection consisted of semi-structured interviews of one to two hours in length, conducted face to face or via telephone with participants recruited from professional development workshops on FASD. The interview questions did not specifically target the topic area of this article, instead they focused on the participants experiences of FASD training, their knowledge of FASD and their professional practices; however, all participants engaged in discussion related to their personal experiences and attitudes as outlined in this article.

Participant responses were recorded and then transcribed and subjected to a process of inductive thematic analysis using the six-step process described by Braun and Clarke [32] (the data presented in this article emerged as a theme from the thematic analysis of a larger body of data, and was separated for dedicated consideration). The researcher (Bagley) kept a record of field notes alongside the interview process to aid in data familiarization and the identification of initial codes, which were subsequently condensed into themes. The overall process was informed by Sandelowski’s widely employed rationale for using qualitative descriptions to generate a picture of professional knowledge [33]. The resulting themes are presented and discussed here, represented by exemplar quotes.

### 2.3. Participants, Materials and Procedures

Thirty-four health, allied health and social and human service professionals participated in this research, including pediatricians, psychiatrists, social workers, neuropsychologists, psychologists, speech language therapists, public health nurses, youth workers, mental health nurses, counsellors, family support workers, child protection workers and public health professionals. Existing studies of professional responses to FASD have tended to focus on single profession cohorts (examples include psychologists [24], nurses [25] and criminal justice professionals [26]), but profession-specific factors were not pertinent to the design of this study, which instead encompassed a wider range of professional viewpoints.

Participants were recruited by the researcher at or after professional development workshops for FASD held throughout New Zealand. These workshops catered to different professional groups but had shared function of awareness-raising for FASD. They served as a meeting point for a wide range of professionals with an interest in furthering their own FASD knowledge. This made them effective recruitment points for this study, as the participants were able to speak from a basis of some degree of prior awareness about FASD, established or reinforced through their workshop participation. The research was undertaken with ethics approval from the University of Otago.

## 3. Results

Four broad themes emerged from the participants’ discussion of, and reflection on, their perceptions of alcohol and risk. Firstly, when considering the factors involved in FASD, participants’ thoughts turned to their own experiences of alcohol consumption and pregnancy. This was particularly the case for participants who were mothers. Secondly, participants described their perceptions of alcohol use in others, whether their partners, friends and acquaintances, or in other social interactions. Thirdly, participants considered FASD in relation to cultural norms around alcohol use and misuse, including discourses on perceived social issues and patterns of social control. Finally, participants compared alcohol to other drugs, raising questions about the perceived links between harm and legality.

### 3.1. Theme 1: Personal Reflections on Alcohol Consumption and Pregnancy

Many of the participants in this study initiated their response to the topic by revealing that they had not been fully aware of the risks associated with consuming alcohol when they (or their partner) were pregnant with their own children. For some, this was in the distant past, but others were more recent parents, as one participant noted:

When I had my first daughter, I thought I could have a drink or two in pregnancy and they would be fine … now, having been to the [FASD] conference, I wouldn’t drink in pregnancy. And I would tell my friends not to. They can make their own choices, but having seen from my very scientific brain, how it affects molecules in the brain, that’s what made the difference for me …

(Psychiatrist)

Other participants expressed an awareness that alcohol consumption during pregnancy was “not ideal” or could “affect the fetus”, but they did not necessarily understand what the actual risks of prenatal alcohol consumption were, as one participant expressed:

When I had my children, I didn’t know about fetal alcohol … the midwives never said anything, the hospital never said anything about it, there was nothing at all … I knew that drinking alcohol during pregnancy was wrong [but] I didn’t have any further knowledge … or that one drink or two drinks could have a huge impact on that baby, I didn’t know at all. I think I just went on my basic knowledge of right and wrong, that you shouldn’t really drink alcohol because it is not that good.

(Social Worker)

Most of the participants were unable to recall, when asked, whether they had been given any formal information about the risks of alcohol consumption during their own (or their partners’) pregnancies. One participant did recall being given a pamphlet, but said that the information it contained was then contradicted by her obstetrician:

He said to me: “Do you have any funny cravings?” And I said: “Oh it’s really weird, I’m really craving beer”, and he laughed, and he said: “Well, if you wanna [sic] have a glass of wine every now and then, that would be fine.” And, I thought, that’s only five years ago.

(Psychologist)

Another participant related the shock she experienced when coming to terms with the disjuncture between conflicting advice on prenatal alcohol consumption, describing the risk as akin to playing ‘Russian roulette’:

You start to look at your own child [and think] “Oh, my God is there [a risk] there? I didn’t know that.” I did drink and I stopped when I got, when I found out I was pregnant, you know. So there’s always that, but I think it’s getting out that knowledge … you know, and that it’s a Russian roulette. Now, if you’re into playing Russian roulette that’s fine, play the game, but that’s what you’re playing and I guess that’s the concept I’d never, never had, as someone wanting to have babies.

(Psychologist)

Reflecting on the issues of prenatal alcohol consumption led some participants to also reflect on their broader drinking habits, as one pediatrician related:

I’m really intrigued actually because I think that in my peer group we all drink too much, and we all [collude] in not thinking about it very hard. That we all accept it, it becomes normal to have a glass a wine most evenings, and it’s cheap and it’s not, you know, it’s not abnormal. And we’ve normalized it to a degree that we don’t stand back and go, actually, you know, 30 years ago people didn’t use to keep wine in their houses, and they didn’t have it every week, they had it [at] Christmas and birthdays and that’s it. And it was normalized enormously, and because we’re drinking it changes it for our kids as well. It’s very normal, even if you’re not getting drunk, you know, I don’t get drunk every night, but I do enjoy a glass of wine, but I’m still drinking.

(Pediatrician)

This was further considered in terms of social expectations of women and drinking, as one participant admitted:

I would have to put my hand up and say I would consume alcohol several times a week, and I think that’s become quite normal. What I mean by that is a couple of drinks sometimes after a hard day at work, social events, that kind of thing, so I think the general consumption of alcohol by women in this country has increased.

(Child Protection Coordinator)

The theme *personal reflections on alcohol consumption and pregnancy* highlighted individual experiences in using alcohol in light of their awareness of FASD. One professional adamantly referred to alcohol use and pregnancy as a major risk to the health of a fetus, akin to playing ‘Russian roulette’. Of interest is the recognition that personal alcohol use was identified as a common experience and a general awareness existed that alcohol use during pregnancy held some risk.

### 3.2. Theme 2: Reflections on Others’ Prenatal Alcohol Consumption

While participants found it confronting to talk about their own alcohol consumption and ignorance of prenatal risk in the research setting, they noted that it was even more challenging to try to have these discussions in social settings, even with friends and family. As one participant noted:

A friend of a friend of mine was pregnant quite recently and I was talking to my friend and I knew she was a bit of a drinker and I said: “Oh, is she still drinking?” And she said: “Yep, she is still drinking”—two units twice a week because she thinks that is OK, and you can’t tell her otherwise, and I kind of didn’t see her because I didn’t want to. I felt quite torn because I knew I would lose a friendship.

(Psychiatrist)

Some participants postulated that the inconsistency in prior knowledge of FASD, and the difficulty of talking to others about alcohol use or misuse, also flowed through their experience as health consumers and also into their social relationships. As one Pediatrician noted:

I think we all just try and keep deluding ourselves. My GP doesn’t like to ask me about my alcohol use. It’s really funny, he doesn’t want to go there, but I think that’s because he drinks like I do, and so he doesn’t want to have to think about it himself … So one of the things, in all the stuff [Alcohol Healthwatch] sent us, there was loads of stuff about assessing people’s drinking habits, and my husband and I sat down with it and went “huh” and [I] immediately went out and bought smaller glasses … we don’t consider ourselves problem drinkers, but we drink very much like an awful lot of our peers, which is more than what the booklet suggests. But it’s just that societal concept that drinking is acceptable isn’t it—it’s the drug of choice.

(Pediatrician)

All of the pediatricians and psychiatrists interviewed (as well as a number of the other professionals) recalled discussions with colleagues who had minimized the risk of alcohol consumption based on their own experiences of alcohol consumption:

Some of my colleagues, you know, have said “Oh, I don’t really like to think about fetal alcohol because you know I think of when I was pregnant and I drank a tiny bit or I drank quite a bit, did that damage my kids? But they kind of turned out all right. Or when my wife was pregnant or my friend …”

(Psychiatrist)

Such attitudes were, according to participants, quite widespread in society, and in the absence of widespread public health prevention campaigns, informed women’s decisions about drinking:

Lots of our people drink through pregnancy, and nothing—there is no noticeable effect on the child that emerges. So you get: “Oh well, I drank with my last two pregnancies and nothing happened so why would it happen now?” Or: “My mother drank through all [her pregnancies]”—and that’s the attitude that you get.

(Social Worker)

One participant more explicitly linked the issue to mixed messages arising in media:

We’ve still got conflicting information coming out on national TV around the use of alcohol. And we’ve still got the perception of it’s not okay to challenge pregnant women if they’re drinking, you know. So we’ve got a society that doesn’t want to address these issues, and that makes it really quite difficult.

(Counsellor)

The theme *personal reflections on others’ prenatal alcohol consumption* revealed a degree of trepidation in participants’ responses. The idea of having a personal conversation with a friend or colleague about alcohol use and pregnancy was both uncomfortable and undesirable, and saying something was perceived to hold the potential risk of losing that relationship. The impact of alcohol use and pregnancy often goes undetected. In the voice of one participant who “immediately went out and bought smaller [wine] glasses”, there was some acknowledgement of potential harm and the need to address personal alcohol use. It is a complex topic that clearly created some discomfort amongst participants personally, and was likely to have an influence on professional practice.

### 3.3. Theme 3: Social Constructions of Alcohol Use and Misuse

Many participants in the study related the communication problems around alcohol and pregnancy to broader social patterns of alcohol use (and misuse). For example:

You hear very little about FAS[D] … because we’re a culture of drinkers and if we start talking about that, people are going to say: “Well, you know bugger you, I want to drink.” There’s no recognition in society that alcohol is probably one of the more damaging drugs that we actually have out there and it’s freely available.

(Social Worker)

Some raised the observation that alcohol misuse is a highly politicized topic in New Zealand, suggesting that alcohol-related harm is minimized in public discourse because of politicians’ own personal views and experiences of alcohol, as well as political and economic pressure on the government by the alcohol industry. For example:

Think about how much the New Zealand government gets from excise duty in a year. I don’t carry this number in my head anymore because it is a horrifying number, and I think it’s in the billions, and if you think the sophistication of the alcohol industry in its marketing initiatives and just how much you can do with money in terms of shifting people’s perceptions …

(Psychiatrist)

Other participants said that New Zealand had progressed some way to highlighting alcohol-related harm in priority areas such as drunk driving, family violence and the impact on the criminal justice system. Participants questioned why there was a reluctance to highlight health risks, especially those related to pregnancy and alcohol use. Participants also observed a tendency in society for alcohol-related harm to be linked to the problem of addiction, and there was a perception by some participants that the other potential risks of social drinking were minimized, whilst addiction was foregrounded. As one pediatrician put it:

I think we’re so fixated on drunk people that we’re just letting all the drinkers who are not drunk kind of swing by, but it’s pretty easy to drink at home and drink more than is good for you without ever feeling drunk or getting into any sort of trouble or mischief, but just quietly drinking more than you should.

(Pediatrician)

Another participant believed there to be a considerable lack of understanding of addiction in the community, and framed the problematization of addiction in terms of oppositional discourses of individual versus social responsibility:

If people feel like you drank in pregnancy, and you have a difficult child, then that’s your problem, not seeing that addiction is, it’s like most mental health [illnesses] … People don’t feel like they need to take a societal responsibility for managing it.

(Psychiatrist)

Some participants thought that the risk of prenatal alcohol consumption crosses social strata, while others postulated that lower socioeconomic communities are at increased risk due to social conventions around drinking and a lack of access to up to date information. One expressed a perception that there is an imbalance between working- and middle-class family educations and knowledge around risk, leading to differentiated health outcomes:

I think the educated young women now don’t drink during pregnancy. I might be wrong, because I’m aware that there’s a whole sort of middleclass of educated women who drink quite heavily too. So I’m not sure about the effects of that. I guess the people I see tend to be at the lower end of the social economic strata …

(Speech Language Therapist)

A child protection professional also noted that FASD may be a particular issue for families in which there were other issues associated with alcohol misuse:

I know that I’m horrified at the number of referrals I get for domestic violence where over-consumption of alcohol is an issue … women are drinking a lot more in a way that they weren’t back a generation or so. Therefore, one has to, I guess, assume that that affects women who are pregnant … So if you live in an environment of alcohol, heavy alcohol consumption, whether it’s with a partner, whether it’s with a family history … I think there are some women who are aware and would give up alcohol, but within those sorts of groupings, I would be cynical about how many. That’s where, I think, the education needs to be targeted.

(Child Protection Coordinator)

The overlapping topics of alcohol, women and social control were discussed by some participants, with some highlighting discourses around women’s rights and equality that need to be considered when discussing maternal alcohol consumption and risk. One participant went as far to consider how such social and legal controls could impact on the rights of women:

It’s a difficult one, I think, because I’m a person who does believe in people’s human rights and the right to make decisions for themselves and to live life the way they want to live it. However, because I work right in the midst of this, I think it’s hard not to be asking the questions of yourself when it comes to children, and unborn children. Who do they actually belong to? Do they actually belong to the woman who’s having them? Do they belong to wider society?

(Child Protection Coordinator)

One male participant considered the role of men within the discourse of women, alcohol and social control:

Are we trying to control women? It’s the whole thing about social medicine as well, so much seems to be about social control and because … we focus so much on in terms of individuals as well … it focuses on women as the problem, and women’s drinking happens in a context: it happens in the context of a society that values alcohol and is permissive around that. How are the men talking and supporting the women around this? I still think, within our cultural framework, it sets women up to be the problem and that makes it really hard for them to actually talk about it, for professionals to ask those questions, for women to actually answer honestly. It positions women as harming their children and women don’t go out intentionally to harm their children.

(Counsellor)

The theme *social constructions of alcohol use and misuse* emerged from participants voicing their concerns about the challenges of a modern, politicized culture of drinking in which industry and government were also implicated. The human toll and health risks associated with social drinking were perceived to be minimized within society, while deep concerns about the harms of heavy drinking were noted including increasing violence in the area of child protection. The use of alcohol during pregnancy raised the issue of women’s autonomy over their own bodies and the rights of fetuses versus the rights of women, and the need exists to recognize how hard it is for women to talk about these issues to professionals, and how difficult it is for professionals to ask questions regarding alcohol use and pregnancy.

### 3.4. Theme 4: Alcohol Compared to Other Drugs

One clear outcome of participant’s reflections on their own experiences with alcohol, and on the social construction of alcohol use, was a readiness to compare the harm cause by alcohol misuse with that of other drugs, legal and illicit. One broadly acknowledged observation arising from this discussion was that society’s perception of risk and harm seemed to be linked to notions of acceptability and legality. As one pediatrician elaborated:

If I went home and smoked a joint every night people would think badly of me, when in actual fact I’d probably do myself less harm … It seems to be more about, um, you know, whether it’s legal or illegal rather than risk. It’s another construct I’m trying to bash away at in the community.

(Pediatrician)

Another participant reflected on how prenatal alcohol consumption might compare to, for example, the harm caused by tobacco:

You know women don’t smoke in pregnancy, well very rarely. And if you see a woman smoking and they are pregnant … it makes [me] feel quite physically sick and I want to go up and say to her: “You shouldn’t be smoking.” But I know that she knows, and so I often don’t, [and] I don’t think we have got there with alcohol in pregnancy. I do see a time when we will be there, but it kind of has to be a gently, gently approach.

(Psychiatrist)

One participant questioned the idea that illegal drugs are more dangerous than alcohol, arguing there is a perception in the community that some types of alcohol pose more risk than other types of alcohol:

Some people would say [that] a glass a wine [or] a glass of beer is not as dangerous as a hard spirit, you know, people might categorize things, oh there’s no harm in a beer, you know people have a beer every night, there’s nothing wrong with a glass of wine, because they drink wine like they do coffee.

(Social worker)

Meanwhile, another participant framed this issue in terms of addiction and culturally biased assumptions about risk, illustrating this using an example of a discussion she had with her doctor when she was pregnant:

He had made a, what’s the word, an assumption on me that I was a white middle class woman who wouldn’t [binge drink] whilst I was pregnant. How does he know that I wasn’t a raving alcoholic, you know and that one glass of wine would actually lead to five? So, I don’t think there’s enough care around the information that’s given. I mean, there’s posters up where you go into the midwives, but you know, they ask you alcohol and drugs in your first [session, and] if you just say no then it’s glossed over, I mean I really don’t think much attention’s paid to it at all.

(Psychologist)

Even in circumstances where FASD was recognized as a health concern, some participants talked about how illicit drug use was often perceived by colleagues as a more serious issue than alcohol use in pregnancy. For two of the participants, this issue came up when providing training to colleagues subsequent to their involvement in the original training project. One medical professional gave an example of such questioning:

He said: “Well, what about other drug use, surely that’s worse?” And I was a bit gob smacked really, and I said: “Well, what is worse? Nothing’s worse than alcohol, really.” Heroin’s not worse, you know, and actually that concept is there, which I think is why the midwives always write down, oh, smoking cannabis and gloss over the alcohol because they get totally hung up on the drugs people are using and don’t think of alcohol as a drug in itself.

(Pediatrician)

The theme *alcohol compared to other drugs* offered insight into the perceptions of legal and illegal substances and their use during pregnancy. One participant identified the concern that white middle class women’s drinking behavior was less likely to be scrutinized by professionals. It is important to note that some participants themselves train other professionals and thus have an opportunity to challenge misperceptions around the harms of alcohol versus drug use in pregnancy. It is clear that most participants recognized the harm of alcohol use in pregnancy. A key message emerging from this theme is that these topics need to be taken up in professional discourse and training in order to ensure health professionals have an accurate understanding of the impact of substance use during pregnancy.

## 4. Discussion

The reflections of participants on their perceptions of alcohol use and risk reveal some significant points for consideration in relation to practice responses to FASD. As a starting point, these reflections make it apparent that professionals, as members of society, are themselves influenced by social norms around alcohol use, by their own attitudes and by personal experiences of alcohol use and pregnancy. Many of the participants in the training session were parents themselves, and related stories about their own experiences of alcohol consumption or those of their spouses, friends and peers. Participants were able to critically self-reflect on this following their participation in professional development on FASD, but they also identified various instances of friends and colleagues engaging in commentary that was not so critical or reflexive, which could lead to mixed messages about harm and risk.

The topic of alcohol use and pregnancy is evocative and despite the discomfort this discussion can raise, people have opinions. Of particular concern is the issue of mixed messages on alcohol use and pregnancy despite increasing awareness of FASD. As Elliott states, “In an era when rates of risky drinking in women continue to rise, when a binge drinking mentality has taken hold of our teenagers, and when almost half of all pregnancies are unplanned, health professionals and teachers can expect to see more children with FASDs” [3] (p. 14). The consequences of mixed messages regarding prenatal alcohol consumption that permeate society in general were described by participants as contributing to unclear or contradictory information. These confusing messages, coupled with a lack of community awareness of the potential risks of alcohol, and other peoples’ uncritical personal views about alcohol use during pregnancy, make prenatal alcohol consumption a difficult topic to broach with clients, but also with friends and family. Marcellus, Poole and Hemsing observed that “substance use during pregnancy is a highly contested space” [34] (p. 213), and is often associated with trauma histories; thus, highlighting the need for an intersectional approach that recognizes the influences of gender, poverty, race, class and housing.

The topics of stigma and marginalization were clearly identified in the results of this qualitative research. At different points, participants talked about risk in relation to factors such as social class and education, substance use disorders, the rights of women to be in control of their own bodies and moral dimensions of legal and illicit drug use. In trying to convey messages about FASD, participants felt that they ran the risk of problematizing their work with clients in raising the issue of alcohol use and pregnancy. The references participants made to illicit drug use are particularly useful to illustrate this point. At the time the research was conducted, New Zealand was experiencing a rise of illicit methamphetamine use (locally called ‘P’) and a growing media-induced wave of moral panic around the increasing incidence of ‘P babies’, that is, babies born with complications arising from their mothers’ prenatal methamphetamine use [35]. While society was alarmed about ‘P’ babies, a lesser response was (and is) applied to prenatal alcohol use, even though the potential for alcohol-related harm was and is considerably more widespread than other drugs. Practitioners may therefore struggle with how to quantify risk in ways that match public expectations. Additionally, the name ‘fetal alcohol spectrum disorder’ is itself loaded with stigma, as it clearly identifies the etiology of the problem. The need exists to reframe FASD as a public health problem that warrants the same supports and services as any other lifelong disability. Professionals have a key role in stigma reduction through FASD-informed practice. This means a solid grounding in knowledge on FASD from a family systems perspective and engagement in trauma-informed practice while building supportive relationships with families and communities [36].

Professionals also recognized that the FASD field is, at this stage, dominated by biomedical frameworks for understanding, and that social science responses in relation to alcohol use or prohibition are rarer. Thus, while it is becoming easier to diagnose and talk about FASD, the act of diagnosis, for example, carries the potential for stigmatizing mothers because of their use of alcohol while pregnant. Professionals are alert to the potential of causing stigma, and may therefore be reluctant to follow through with certain actions (as described directly by the participant who chose to avoid her alcohol-consuming pregnant friend). It is likely that these responses impact not only individuals, families and women in general, but also contribute to a general reluctance by professionals to become engaged with FASD work in the first place. In some cases, participants speculated that some health professionals were unwilling to consider FASD because it is too controversial, or that professionals in general were reluctant to engage with FASD because of the normalization of alcohol in New Zealand society. This societal positioning, combined with the research participants’ own personal experiences of alcohol (and in some cases alcohol consumption during their own pregnancies), may indeed have influenced or contributed to a reluctance to consider FASD in their professional practice.

### Limitations

The scope of investigation for this research was limited to New Zealand, and the perspectives provided reflect just a moment in time, not an ongoing investigation. However, many of the New Zealand-specific comments have parallels in other communities given that FASD is a global problem, and thus has the potential to be understandable and relatable to professionals elsewhere. The participants in this study were recruited from FASD professional development sessions and thus possibly had a greater interest in FASD compared to professionals in general. The knowledge gained in these professional development sessions may also mean that the study participants had greater knowledge of FASD compared to other professionals, and that they may have been more self-motivated to participate in the study. This recruitment process had the advantage of being relevant to the needs and knowledge of the participants, but it was also limited in that it did not include the voices of professionals not actively engaged in seeking knowledge about FASD, and whose perspectives might sit in contrast to the discourses presented here.

## 5. Conclusions

This research has demonstrated that, when considering alcohol consumption and the risks related to FASD, professionals refer to multiple ‘non-clinical’ frames of reference. In this instance, the four main frames of reference were: reflection on personal experience; experiences of friends, relatives and acquaintances; social constructions of alcohol use and misuse; and comparisons to other types of drug use. Each of these frames of reference presented mixed messages about harm and risk that contradicted the participants’ professional knowledge of FASD, yet they form part of the decision-making environments in which participants conduct their professional practices.

In general, the overall impression provided by these discussions is that participants often viewed the problems associated with alcohol consumption as being beyond their ability to control or even begin to approach. These comments reveal that health and allied health professionals’ own experiences, views and opinions of alcohol and drinking culture play a part in how they conceptualize an issue like FASD. Many of them clearly have the capacity to imagine themselves in a position of harm or risk, despite their relatively high levels of education, income and professional status. This dispels the perception that FASD affects only socially and economically marginalized segments of society. However, this empathy also revealed a level of insecurity in regard to how to broach the topic of prenatal alcohol consumption with peers. In the absence of specific models of practice, the need exists for specific clinical training models that support professionals to explore their own attitudes and beliefs regarding alcohol use, while guiding a professional response grounded in current research and best practice in FASD prevention and intervention. It is important in professional training in relation to FASD, alcohol use and pregnancy to make space to talk about the discomfort this topic raises. Above all, growing rates of FASD demand that professional practice rise above potential personal views and offer a clear pathway for guidelines for clinical intervention.

These findings are significant to professional practice regarding FASD because, while professional knowledge of and services for FASD remain inconsistent or inexistent, non-clinical factors are relied upon to help fill the knowledge gap that exists. This may result in professionals stepping back from engaging with FASD in the absence of FASD training or clear diagnostic or intervention practice guidelines, or because they fear exacerbating other kids of social harm through unintentionally causing stigma or marginalization. However, on a positive note, participants were able to clearly articulate knowledge growth and improved understanding of FASD compared with their prior knowledge or awareness about the effects of prenatal alcohol consumption. This reflexivity has the potential to counterbalance the participants’ own concerns about engaging with FASD in their practices. Further research into practitioner attitudes towards FASD may benefit from an extended focus of reflective practice that enables practitioners to deconstruct and make sense of the cultural, personal and societal factors that permeate in discussions of alcohol-related harm and risk.
